# Veterinary Surgical Notes

**Published:** 1882-04

**Authors:** 


Veterinary Surgical Notes.—Among German veterinarians the use of a red-hot iron in diseases of the joints and of bones has had very happy results. Dr. Utz recommends highly the use of the needle in the colic of horses. The rumen of cows, according to Gotz, should not be punctured when the animal is advanced in pregnancy. Dropsy of the scrotal sac in oxen is very radically cured by simply cutting off the scrotum. Broken legs in calves can be cured, according to Ow, by the use of plaster-of-Paris, or by a simple clay bandage. A cure for cancer in the lower animals is said to be the application of oil of turpentine to the affected surface; this being covered with oakum and firmly held down by a bandage.

